# Benefits of resistance training on body composition and glucose clearance are inhibited by long-term low carbohydrate diet in rats

**DOI:** 10.1371/journal.pone.0207951

**Published:** 2018-12-07

**Authors:** Uliana Sbeguen Stotzer, Graziéle Fernanda Deriggi Pisani, Gustavo Henrique Rigo Canevazzi, Gilberto Eiji Shiguemoto, Ana Cláudia Garcia de Oliveira Duarte, Sergio Eduardo de Andrade Perez, Heloisa Sobreiro Selistre-de-Araújo

**Affiliations:** 1 Department of Physiological Sciences, Federal University of São Carlos, São Carlos, SP, Brazil; 2 Department of Physical Education and Human Motricity, Federal University of São Carlos, São Carlos, SP, Brazil; Max Delbruck Centrum fur Molekulare Medizin Berlin Buch, GERMANY

## Abstract

**Background/Objectives:**

Regular exercise training is effective to altering many markers of metabolic syndrome and its effects are strongly influenced by the type of consumed diet. Nowadays, resistance training (RT) has been frequently associated with low-carbohydrate high-fat diet (LCD). After long term these diets causes body weight (BW) regain with deleterious effects on body composition and metabolic risk factors. The effects of RT associated with long-term LCD on these parameters remain unexplored. We aimed to investigate the effects of RT when associated with long-term LCD on BW, feed efficiency, body composition, glucose homeostasis, liver parameters and serum biochemical parameters during BW regain period in rats.

**Subjects/Methods:**

Male *Sprague–Dawley* rats were fed with LCD (LC groups) or standard diet (STD) (ST groups). After 10 weeks-diet animals were separated into sedentary (Sed-LC and Sed-ST) and resistance-trained (RT-LC and RT-ST) groups (N = 8/group). RT groups performed an 11-week climbing program on a ladder with progressive load. Dual x-ray absorptiometry, glucose tolerance tests and insulin tolerance tests were performed at weeks 10 and 20. Liver and serum were collected at week 21.

**Results:**

RT reduced feed efficiency, BW gain, liver fat and total and LDL cholesterol, and improved body composition and glucose clearance in animals fed on STD. In those fed with LCD, RT reduced caloric intake, BW regain, liver fat and serum triglycerides levels. However, improvement in body composition was inhibited and bone mineral density and glucose clearance was further impaired in this association.

**Conclusions:**

The LCD nullifies the beneficial effects of RT on body composition, glucose homeostasis and impairs some health parameters. Our results do not support the association of RT with LCD in a long term period.

## Introduction

Metabolic syndrome is a very common cardio-metabolic risk condition related to physical inactivity, overweight and obesity [1,2]. Exercise and diets have proven be effective at beneficially altering many markers of metabolic health, including abdominal adiposity, insulin sensitivity, and blood lipid profile [1]. Regular exercise training is effective to prevent and treat several diseases [1,3,4]. While both aerobic and resistance training (RT) favors insulin sensitivity, glucose uptake and serum biochemical parameters [3,5], aerobic training is associated with greater body weight (BW) loss; on the other hand, RT is associated with increases in muscle mass and strength. RT improves mainly body composition, even without changing BW, due to increase in skeletal muscle mass and decrease in fat mass (FM) and visceral adipose tissue (VAT) [5–7], a deposit strongly and positively correlated with morbidity and mortality [8,9].

The effects of exercise training are strongly influenced by the type of consumed diet [10], making the association of exercise and specific diets to become very popular [9,11]. As a rule, weight recovery usually occurs after long term diets [11,12]. Lean individuals that try to lose weight on some form of diet are more prone to this recovery [12] that occurs with a faster rate of FM recovery relative to free-fat mass (FFM) recovery [11]. The association of exercise training and diet can be a good strategy for long-term obesity management [10] with potent benefits on body composition [4,11]. Among the different types of exercises, RT is indicated since it prevents visceral fat regain and reduce BW regain [13], increases lean body mass and maintains abdominal adipose tissue depots, homeostasis model assessment (HOMA), and insulin-sensitivity check index (QUICKI) [1] during weight regain period in humans.

Low-carbohydrate high-fat diets (LCD) are increasingly being used to promote rapid BW loss [14,15]. Nevertheless, difference of BW loss is attenuated over time in long-term studies, conducted in mice [16] and humans [15,17] (long-term: ≥ 12 months for clinical studies [10,14] and usually ≥ 8 weeks for rodent studies [16,18,19]. After long-term feeding LCD, mice show BW regain and impairments in body composition and glucose tolerance [16]. Returning to a standard diet (STD) after 8 weeks consuming a LCD, rats increased BW gain, adiposity and insulin levels [20].

Free-fat mass loss and FM increase are a potential concern of LCD [19,21–23], mainly in long-term studies. Despite the role of RT in maintain FFM and of this association with LCD being largely used by humans, a limited number of studies have examined the effects of this association. In elderly men, 10 weeks of RT associated with LCD had similar effects on FFM compared to RT associated with a STD [24]. In overweight women, RT associated with LCD carried out for 12 weeks reduces FM without altering FFM, while the group that followed a STD showed increased FFM without changes in the FM [22]. Another study performed in LCD-fed rats fed over 6 weeks showed that training in a continuous resisted voluntary wheel-running did not impair acute or chronic skeletal muscle hypertrophic responses [25] and no differences to the white adipose tissue mass or liver triglycerides (TGL) were found, compared to the control diet [26]. However, it is important to note that these studies had a short or a medium duration and, to the best of our knowledge, the effects of RT on body composition and metabolic risk factors when associated to long-term LCD, remain unexplored.

Therefore, the aim of this study was to investigate the effects of RT on BW, feed efficiency, body composition, glucose homeostasis, liver and serum biochemical parameters, during the BW regain period of long-term LCD-fed rats. We used this experimental approach of RT since there is evidence that it improves body composition, glucose levels, serum lipid profile, hepatic lipid metabolism, skeletal muscle hypertrophy and adipose tissue in models of diet- and ovariectomy-induced obesity in rats [27–33]. However the effects of RT when associated with LCD in long term remain unknown.

## Materials and methods

### Animal

All animal procedures were conducted in accordance with the Brazilian National Guidelines for the Care and Use of Animals [34] and were approved by the Committee of Experimental Animals from the Federal University of São Carlos (2932120515) and all efforts were made to minimize animal suffering. Male *Sprague–Dawley* rats (N = 32, 135.2 ± 15g, 5 weeks old, from State University of São Paulo, Araraquara, Brazil) were housed in polypropylene cages (3 rats per cage) at a constant temperature (22 ± 2°C), following a 12-h light/12-h dark cycle (lights on at 7:00 pm). Access to water and food were *ad libitum* throughout the experimental period. After the 21-week experimental period and 48h after the last training session, in order to obtain samples in the state of accumulated training effect, rats were fasted (about 6 h) before liver and serum were collected for posterior analysis.

### Groups and diets

After 1 week of acclimation, rats were divided into two weight-matched groups. Animals on ST groups were fed with the AIN-93M STD— (3.85 kcal/g—9% calories from fat, 76% from carbohydrate, and 15% of protein– 3.85 kcal/g) [35] and those on LC groups were fed with a modified AIN-93M diet to a moderate LCD (5.41kcal/g—59% fat, 31% carbohydrate, 10% protein), for 21 weeks (10-week diet pre-RT period plus 11-week RT period). These diets are suitable for chronic rat studies [35] and reach the nutritional requirements [36]. They were prepared by Prag Soluções (Jau, SP, Brazil).

**Fig 1:**
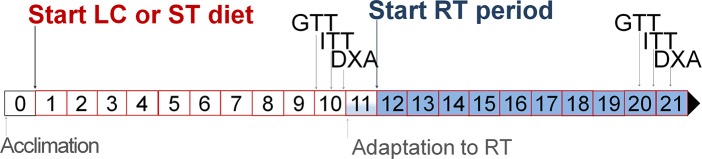
Experimental procedures conducted over the weeks of experiment.

After a 10-week diet, each group was weight-matched and subdivided into sedentary (Sed) or resistance-trained (RT), forming the following 4 groups (N = 8/group): Sed-ST, RT-ST, Sed-LC and RT-LC groups. Sed groups were kept in their cages throughout the duration of the experiment without any kind of exercise, whereas RT groups performed an 11-week course of RT, starting at week 10 of diet (Fig 1).

### Resistance training

The progressive RT protocol was adapted from Hornberger and Farrar [33], as previously described [29]. Briefly, the protocol was performed in the dark phase, and it was performed 3 times/week, which the animals were required to climb a vertical ladder (1.1m, 80^0^ incline) with weights attached to their tails until reaching a housing chamber at the top of the ladder, where they were allowed to rest for 120s. Before performing the maximum workload test, the rats were first adapted to the RT protocol for one week. The training sessions consisted of 4 ladder climbs with weights that were 65, 85, 95, and 100% of the rat’s maximum workload, followed by climbs with 30g loads added until the load would not allow the animals to successfully climb to the housing chamber. The highest load successfully carried up the entire ladder was considered to be the maximum workload for that training session and was thereafter used in the next training session.

### Food and caloric intake, body weight and feed efficiency

Body weight and food intake were monitored 3 times/week. Feeding efficiency was calculated as grams of the body mass gained per gram of food intake, per week.

### Body composition

Body composition analysis was carried out at weeks 10 and 20 of the experimental period, using a Dual-Energy X-ray Absorptiometry scan (DXA; Discovery QDR* series, Marlborough, MA, USA). Rats were anaesthetized using a mixture of ketamine–xylazine (32–6 mg.kg^-1^) and were placed in a prone position for the scanning. Total and visceral FM percentage, lean mass percentage (not considering bone mineral content), appendicular skeletal mass (the sum of the lean tissue in the upper and lower limbs), and total bone mineral density (BMD) were calculated. Percentages were calculated as the amount of fat or FFM normalized to BW at the time of measurement.

### Liver parameters

The percentage of lipid content in the liver was determined by the gravimetric method [7] and glycogen quantification by colorimetric method [37], as previously described.

### Biochemical serum analyses

Serum levels of TGL, total cholesterol, low density lipoprotein (LDL), and high density lipoprotein (HDL) from blood collected at week 21 and measured using commercially available kits (Bioclin, Belo Horizonte–MG, Brazil) in an automated plate reader (SpectraMax i3x machine, Molecular Devices, California, USA).

### Glucose tolerance (GTT) and insulin tolerance (ITT) tests

After 10 and 20 weeks of the experimental period, rats were fasted for 6h before an intraperitoneal injection of glucose (1.5 g/kg), for the GTT test, or insulin (0.75 U/kg), for the ITT test. Blood samples were obtained from the tail vein at baseline and at 15, 30, 60, 90, and 120min after glucose challenge for the GTT; and at baseline and at 15, 30, 45, and 60min after insulin injection for the ITT, using commercially available glucose tests trips and a glucometer (Accu-Check, Roche Diagnostic, Indianapolis, USA). An interval of 5 days was provided between the two tests.

### Statistical analyses

After normality (Shapiro-Wilk) and the homoscedasticity (Bartlett criterion) tests, data were compared using the ANOVA test. Dates from pre-RT period were analyzed with ANOVA one-way and those from the RT period with ANOVA two-way (intervening variables week vs. RT). When the group mean had p value ≤0.05, post-hoc analysis (Tukey’s test) for multiple comparisons was performed. Data are presented as mean ± SD. Statistical analyses were performed using the SigmaPlot software 12.0 (Systat Software, Chicago, IL).

## Results

### Food and caloric intake, feed efficiency, body weight and maximum workload

**Fig 2:**
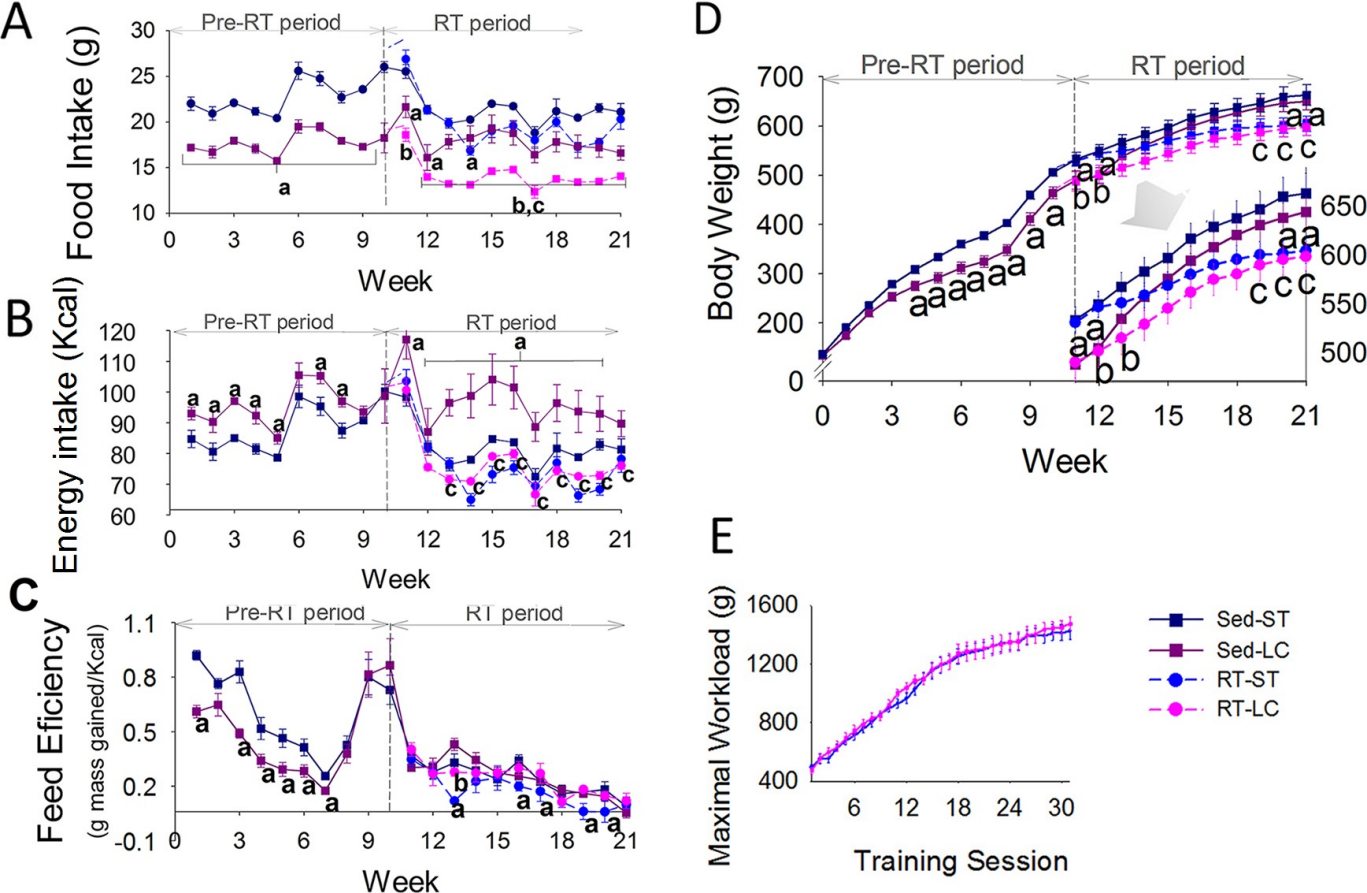
Effects of RT on food intake. (A) Food Intake, (B) Caloric Intake, (C) Feed Efficiency, (D) Body Weight, and (E) Maximum Workload. N = 8/group. ^a^p ≤ 0.05 vs. Sed-ST; ^b^p ≤ 0.05 vs. RT-ST and ^c^p ≤ 0.05 vs. Sed-LC.

In the pre-RT period, animals fed on LCD had lower food intake and feed efficiency and higher caloric intake than those fed on STD (Fig 2A–2C). RT decreased food intake in the LC group and its caloric intake became similar to the ST groups. Feed efficiency was lowest in the RT-ST group. In the pre-RT period, LC group had lower BW. From week 13, the BW of the Sed-LC group became similar to the Sed-ST and from week 19 became greater than the RT-LC group. Trained groups had similar BW from week 14. At the end of the RT period, trained animals had lower BW than Sed animals (Fig 2A–2D). RT groups presented similar and progressive increase in maximal workload throughout the RT period (Fig 2E).

### Body composition

**Fig 3:**
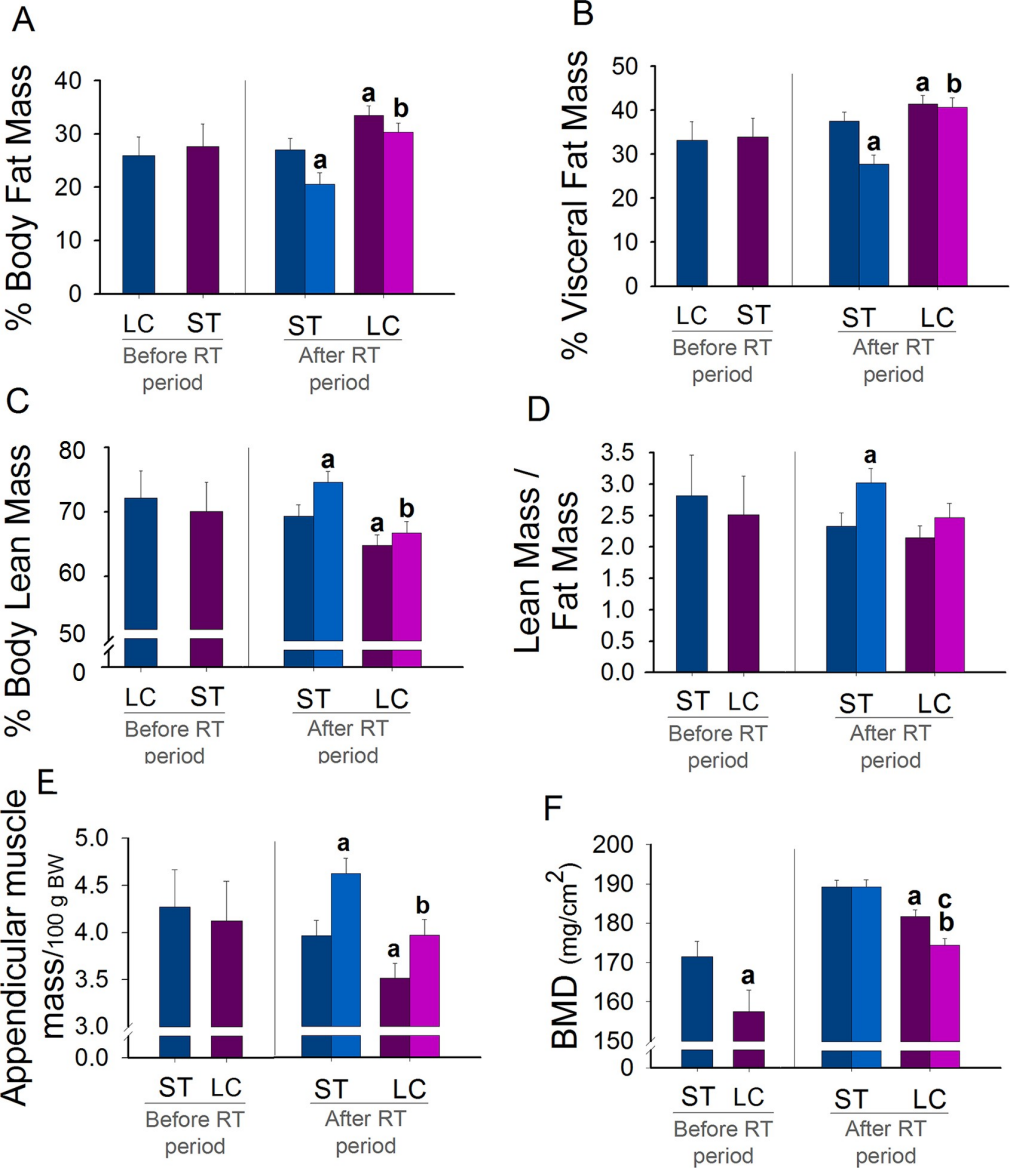
Effects of RT on body composition (DXA) associated with different diets. % (A) Body Fat mass, (B) % Visceral Fat Mass, (C) % Body Lean Mass, (D) Lean Mass/Fat Mass, (E) Appendicular Muscle Mass, and (F) BMD. N = 8/group. ^a^p ≤ 0.05 vs. Sed-ST ^b^p ≤ 0.05 vs. RT-ST and ^c^p ≤ 0.05 vs. Sed-LC.

Before starting the RT period, ST and LC groups had similar body composition, except for BMD, that was lower in LC animals. After the RT period, RT decreased body and visceral FM percentage and increased lean and skeletal muscle mass percentage and lean to FM ratio in the ST animals. In animals fed with LCD, RT did not alter body composition, with exception for BMD that was lower in RT animals. These groups fed with LCD (Sed-LC and RT-LC) had higher body and visceral FM percentage and lower lean and skeletal muscle mass percentage and BMD compared to their respective ST groups (Sed-ST and RT-ST) (Fig 3A–3F).

### Liver parameters

**Fig 4:**
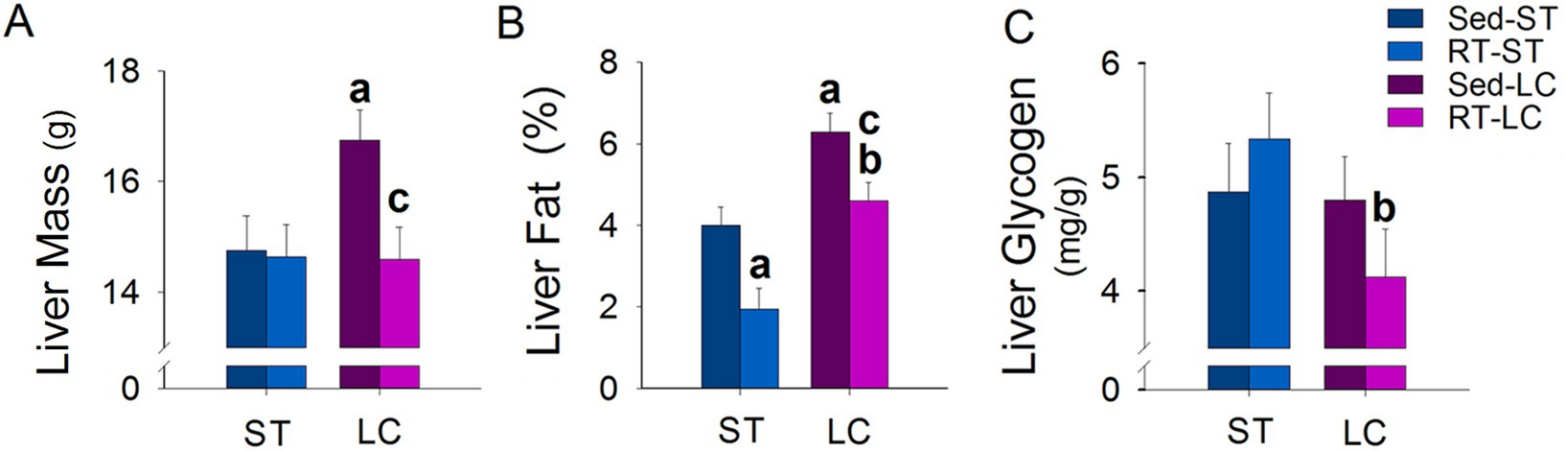
Effects of RT on liver parameters. (A) Liver mass, (B) Liver fat, and (C) Liver glycogen. N = 8/group. ^a^p ≤ 0.05 vs. Sed-ST; ^b^p ≤ 0.05 vs. RT-ST and ^c^p ≤ 0.05 vs. Sed-LC.

The Sed-LC group had the highest liver mass and relative fat mass in the liver. The RT groups (RT-ST and RT-LC) had lower fat percentage than their respective Sed groups (Sed-ST and Sed-LC). Concerning RT groups, those fed on LCD had higher fat and lower liver glycogen deposits (Fig 4A–4C).

### Biochemical serum analysis

**Fig 5:**
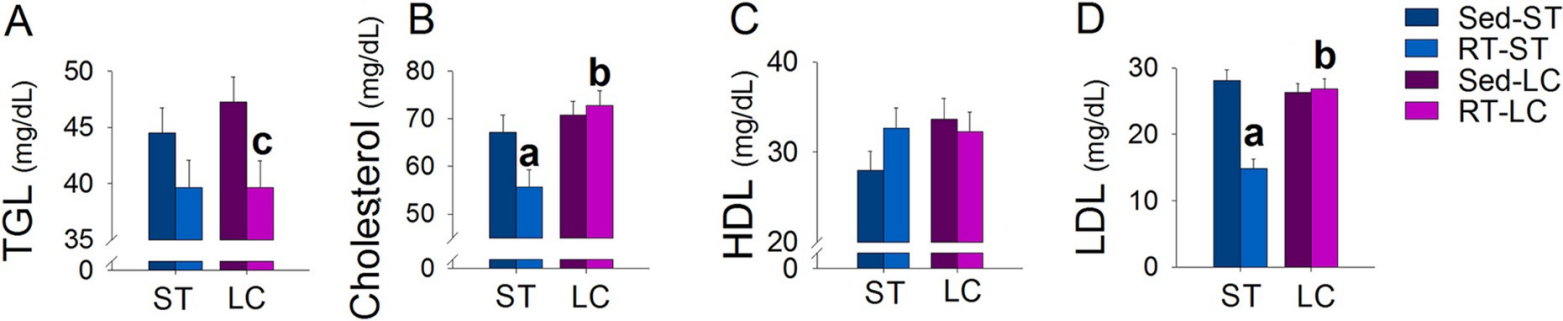
Effects of RT on serum biochemical parameters. (A) TGL, (B) Total Cholesterol, (C) HDL, and (D) LDL. N = 8/group. ^a^p ≤ 0.05 vs. Sed-ST; ^b^p ≤ 0.05 vs. RT-ST and ^c^p ≤ 0.05 vs. Sed-LC.

The RT decreased total and LDL cholesterol levels in the ST group and TGL in the LC group. Among RT groups, LC had higher total and LDL cholesterol (Fig 5A–5D).

### Glucose tolerance and insulin resistance tests

**Fig 6:**
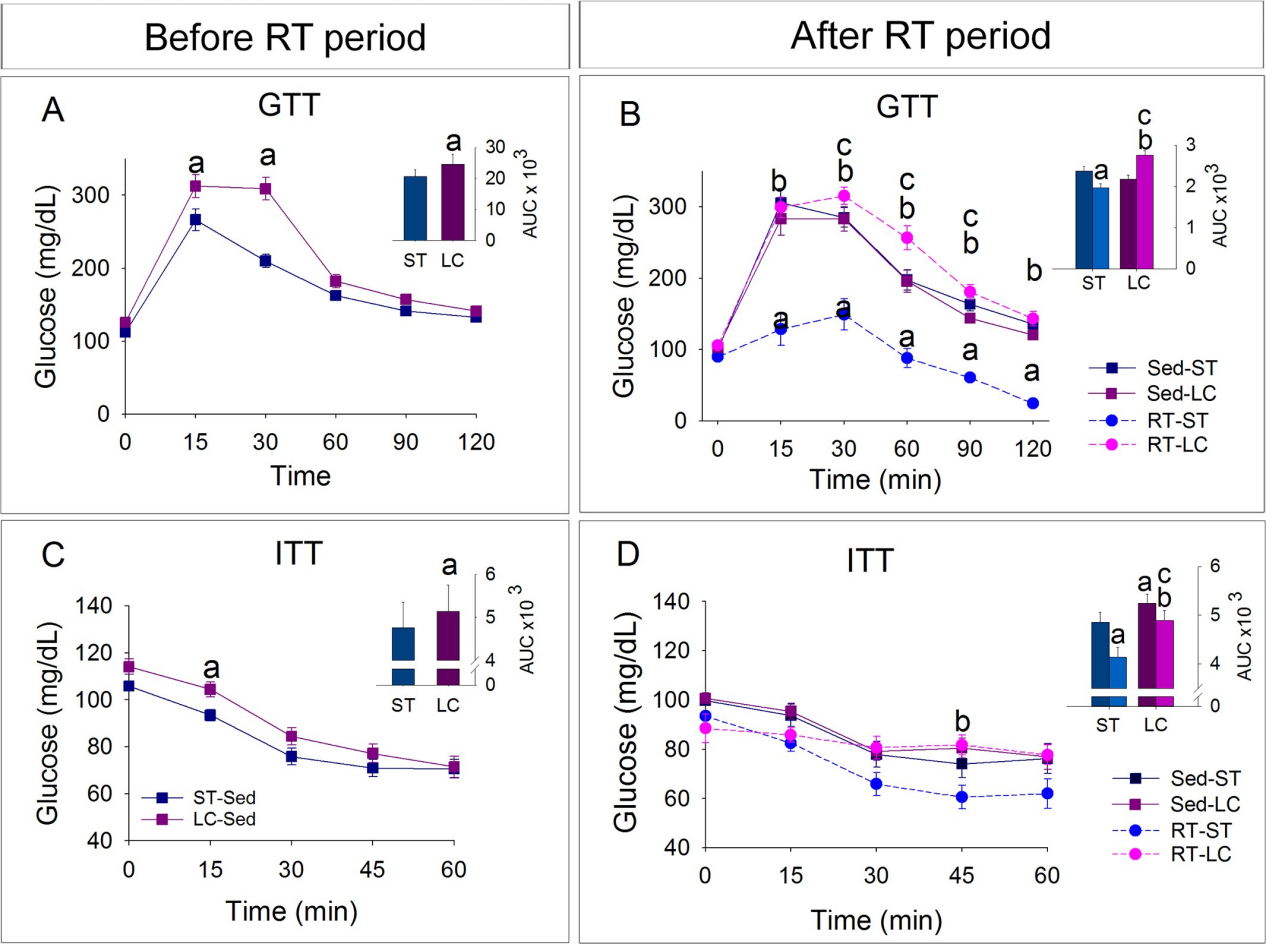
Effects of RT on glucose homeostasis. Glucose (GTT) or Insulin (ITT) tolerance tests and the respective area under the curve (AUC). N = 8/group. ^a^p ≤ 0.05 vs. Sed-ST; ^b^p ≤ 0.05 vs. RT-ST and ^c^p ≤ 0.05 vs. Sed-LC.

During GTT, before starting the RT period, LC rats showed higher glucose levels (15’and 30’) and area under the curve (AUC) compared to ST animals (Fig 6A). After the RT period, sedentary groups had similar glucose levels. The RT increased glucose levels (30’- 90’) and AUC in the LC animals while in ST animals, RT decreased glucose levels (15’-120’) and AUC (Fig 6B).

During ITT, before the RT period, LC animals showed higher AUC than ST animals and decrease in glucose levels (0’-30’) was similar between these groups (29% and 31%, respectively) (Fig 6C). After the RT period, LCD-fed groups had higher AUC than respective ST-fed groups. The RT-ST group had lower AUC and a greater decrease (-30%) in glucose levels (0’- 30’) than Sed-ST animals (-23%). In animals fed on LCD, RT decreased AUC but glucose falls following the insulin bolus was impaired (-11% in trained and -20% in sedentary animals) (Fig 6D).

## Discussion

Our research was motivated by the lack of information regarding the metabolic effects of RT when associated with LCD in long periods and our results provide a comprehensive examination in rats. To the best of our knowledge, RT effects were still not addressed in this experimental condition. The benefits of RT on BW and body composition were inhibited when RT was associated with long-term LCD. Also, glucose tolerance, insulin sensitivity and BMD worsened with this approach.

The BW of Sed-LC group varied similarly to other previous studies of long-term LCD both in rodents [16] and human [15,38]. The initial and transient reduction in feed efficiency caused the lower BW gain initial observed in LC group, even with a higher caloric intake. After long-term diet the expected [11–13] BW regain was observed in Sed-LC group. RT prevented BW regain in LC animals by decreasing food intake and liver glycogen deposits, which also reduces water content [17]. Despite similar caloric intake between RT groups, the lower BW gain of that fed on STD is due to the decreased feed efficiency, which is related to the increased lean mass percentage, a known effect of RT [5]. Albeit RT prevented LCD-induced decreases in skeletal mass, a concern of LCD both in humans [21] and rodents with higher protein content [39], hypertrophy was completely abolished in animals fed on LCD, results similar to those found after 30 weeks of LCD in a study that used a model of muscle overloading [40]. These results suggest that long-term LCD impairs the hypertrophic response to RT. A LC/High protein diet shows similar hypertrophic response to a Western diet (HC/HF) [25].

Increased adipose mass found after long-term LCD was previously demonstrated in animals with lower BW fed on a severe carbohydrate restrict diet in short-term [39,41,42]. The main effect of RT on body composition is a shift from fat to muscle mass [5,6]. Although RT has the potential to maintain VAT during weight regain [13], as RT did not increase lean mass in LCD-fed animals, the beneficial effects of RT on body and visceral FM percentage were inhibited. Trained animals fed with long-term LCD exhibited increased body fat and decreased lean mass, a known effect of diets in long term [11,12]. Our results showed that RT lost its efficiency of improving body composition when associated with LCD.

Excess visceral adiposity is closely related with ectopic fat [8]. Intracellular accumulation of lipids in muscle and liver triggers the activation of protein kinase C with subsequent impairment in insulin signaling [43]. Steatotic liver, as we found in LC groups, is resistant to insulin and inhibit hepatic glucose production and stimulation of glycogen synthesis [43]. High intensity exercise regulates glucose and lipid metabolism in the liver of rats fed on high-fat diet [44] and promotes glycogen supercompensation [32]. While liver glycogen tended to increase in response to RT in STD-fed animals, although not reaching significance, it was reduced in those fed LCD, suggesting that the LCD minimized the glycogen repletion in response to the hepatic glycogenolysis [45] promoted by training.

Liver is the major regulator of metabolite flow in the body with the ability for lipid uptake from circulation and the release of their products at a moderated rate [46]. Exercise can improve lipid profile, with exercise volume being the most important variable [47]. In the liver of OVX rats RT increases lipid oxidation and reduced lipogenesis [28], effect that may have contributed to decrease liver lipids of both trained groups. On the other hand, RT was not able to for decrease lipid content in circulation when associated with LCD, even both groups having similar volume of exercise. Beneficial effects of RT on total and LDL cholesterol found in animals fed on STD can also be related to weight loss [4].

Impaired body composition increases the obesity-induced changes of developing type 2 diabetes [8,48]. Reduced insulin-stimulated glucose uptake after long-term LCD at least partly, was due to the decreased skeletal muscle mass, which is responsible for the majority of whole-body insulin stimulated glucose disposal [48]. Accumulation of intramyocellular lipids and triglyceride in muscle of rodents fed on LCD was also demonstrated contribute to insulin resistance [39]. On the other hand, intense exercise can improve the insulin-stimulated glucose uptake [45],effect observed in the RT-ST group that is related to the increased lean mass. In contrast, RT-LC group had worse glucose clearance following both GTT and ITT tests, even compared to Sed-LC group that had similar lean mass. These antagonistic effects to those found in animals fed on STD suggest that RT may have been more strenuous for the LC-fed animals, since exercise-induced muscle damage impairs insulin-stimulated uptake of glucose [49], despite maximal workload was similar for both trained groups. Similar muscle glycogen (data not showed) and reduced TGL in RT-LC group is also indicative of muscle damage, suggesting a compensatory lipid utilization [49].

Insulin resistance in osteoblasts reduces osteoblastogenesis, which contributes to impair whole-body glucose homeostasis by reducing osteocalcin, a hormone that stimulates inulin production in pancreas [50]. Decreased BMD found in Sed-LC group is according to a previous study in male rats [51]. As the RT-LC group had the worst sensitivity to insulin in muscle and liver we suspect that the additional BMD impairment found in RT-LC group was due to a bone-specific insulin resistance. Future studies need to address this question on bone within this condition.

To the best of our knowledge, this is the first study assessing RT effects in association with long-term LCD on body composition, metabolic, and liver parameters. The major limitation of this study was the lack of analysis of insulin resistance in specific tissues. However, our aim was to perform an endocrine approach. As future perspectives, adaptations within organs and tissues in response to long-term protocol may be investigated.

In conclusion, although RT prevented long-term LCD-induced BW regain, LCD inhibited RT benefits on body composition and glucose clearance. Our results suggest that association of RT with long-term LCD maybe deleterious for human health and therefore this association should not be indicated.

## Supporting information

S1 Supporting Information Raw data. (XLSX)
